# Prevalence of Low Back Pain in Nonworking Women in Eastern Saudi Arabia: A Cross-sectional Survey

**DOI:** 10.7759/cureus.101484

**Published:** 2026-01-13

**Authors:** Zahra Alhasan, Ali M Al Mousa, Hassan M Alturaiki, Othman Altaissan, Abdullah H Alramadan

**Affiliations:** 1 College of Medicine, King Faisal University, Al Hofuf, SAU; 2 Internal Medicine, King Fahad General Hospital, Jeddah, SAU; 3 Emergency Medicine, King Faisal University, Al Hofuf, SAU; 4 Neurosurgery and Spine Surgery, Qatif Central Hospital, Qatif, SAU

**Keywords:** disability, female, low back, pain, saudi arabia

## Abstract

Background

Low back pain (LBP) is one of the most common public health, economic, and social problems worldwide. LBP has been reported to be more prevalent in nonworking populations than in working populations among both men and women. Therefore, this study aimed to evaluate the prevalence of LBP among nonworking women in the Eastern region of Saudi Arabia.

Methods

A cross-sectional study was conducted in the Eastern region of Saudi Arabia from October to November 2023 using an online questionnaire.

Results

A total of 390 eligible participants were included. The prevalence of LBP among nonworking women was 92.1%. Age was the only factor significantly associated with LBP (p = 0.034). Other factors showed no significant association, including education level (p = 0.954), marital status (p = 0.061), socioeconomic status (p = 0.305), number of children (p = 1.000), pregnancy status (p = 0.135), smoking (p = 0.576), frequency of exercise (p = 0.118), and body mass index (BMI) (p = 0.096). Additionally, most participants (76%) reported that LBP never prevented them from performing their usual daily activities.

Conclusion

This study demonstrates a high prevalence of LBP among nonworking women, with age identified as the only significant associated factor. Despite this high prevalence, LBP did not appear to substantially interfere with participants’ ability to perform their usual daily activities.

## Introduction

Low back pain (LBP) is a major global health problem and a leading cause of years lived with disability worldwide. Its burden continues to increase due to population growth and aging, resulting in substantial social and economic consequences, including reduced workforce productivity [1-3].

LBP is the most prevalent chronic pain and spinal condition, affecting a large proportion of individuals at some point in their lives [4]. It is a leading contributor to disability, a common reason for healthcare utilization, and a major cause of work absenteeism in many countries, including the United States [5-7]. Globally, LBP primarily affects adults, with prevalence increasing during middle age. Although women generally report higher rates of LBP, gender-related differences are not consistent across studies, and a greater impact has been observed in households with nonworking women compared with those with employed women [8].

Previous research on LBP has predominantly focused on working populations, such as office employees, military personnel, and healthcare workers [9-14]. Consequently, the burden and characteristics of LBP among nonworking individuals remain insufficiently explored. This imbalance limits a comprehensive understanding of LBP across population groups and overlooks an important and under-researched segment of society.

This study addresses this gap by focusing on nonworking women in the Eastern region of Saudi Arabia. Understanding LBP in this population is essential, as its prevalence and impact may differ from those observed in working populations. Therefore, this study aims to evaluate the prevalence of LBP among nonworking women in the Eastern region of Saudi Arabia.

## Materials and methods

A cross-sectional study was conducted in the Eastern region of Saudi Arabia from October to December 2023. Data were collected using an online questionnaire distributed via social media platforms, including WhatsApp, Instagram, Telegram, and X. The inclusion criteria comprised Arabic-speaking, nonworking Saudi women aged 18 years or older residing in the Eastern region. Nonworking status was self-reported by participants. Individuals younger than 18 years, men, working women, non-Saudi citizens, those who declined participation, and respondents who did not complete the questionnaire were excluded.

The research instrument was developed through a structured process that included a preparatory phase followed by a final revision by an expert spine surgeon certified as a consultant. During the preparatory phase, an extensive literature review was conducted, including published studies on the prevalence of LBP among nonworking women globally. Based on this review, a questionnaire was designed to assess the prevalence of LBP and its associated risk factors. The questionnaire was written in clear, straightforward Arabic. Content validity was assessed by the expert to ensure relevance, accuracy, and appropriateness. The final version of the questionnaire consisted of 32 items divided into four sections: demographic characteristics, risk factors associated with LBP, characteristics of LBP, and associated neurological symptoms. Body mass index (BMI) was calculated using the standard formula (weight in kilograms divided by height in meters squared, kg/m²) and categorized according to the World Health Organization (WHO) criteria as underweight, normal weight, overweight, or obese.

Data analysis was performed using IBM SPSS Statistics for Windows, version 26.0 (IBM Corp., Armonk, NY, USA). Descriptive statistics were used to summarize the data in frequency tables. Inferential analysis was conducted using the chi-square test to compare variables. A p-value < 0.05 was considered statistically significant.

## Results

The electronic questionnaire received a total of 685 responses. Of these, 390 participants were eligible for inclusion based on the study’s inclusion and exclusion criteria, while 43 responses were excluded due to incomplete questionnaires, resulting in an acceptance rate of 56.94%.

Sociodemographic profile

As shown in Table 1, the demographic analysis of nonworking women in the Eastern region of Saudi Arabia revealed that the largest proportion of participants (42.1%) were aged 36-50 years. Most participants were married (82.1%). Regarding educational attainment, 52.1% held a university degree, while 41.0% had completed pre-university education.

**Table 1:** Demographic characteristics of nonworking women in the Eastern region, Saudi Arabia

Variable	Category	N (%)
Age	<25	55 (14.1%)
	26-35	117 (30%)
	36-50	164 (42.1%)
	>50	54 (13.8%)
Education	Postgraduate	17 (4.4%)
	University	203 (52.1%)
	Pre-university	160 (41%)
	No education	10 (2.6%)
Marital status	Single	50 (12.8%)
	Married	320 (82.1%)
	Divorced	10 (2.6%)
	Widowed	10 (2.6%)
Income	<5000 SAR	186 (47.7%)
	5000-10,000 SAR	133 (34.1%)
	>10,000 SAR	71 (18.2%)
Number of children	0	70 (17.99%)
	1-3	131 (33.68%)
	4-6	155 (39.85%)
	7-10	33 (8.48%)
Current pregnant	Yes	18 (4.6%)
	No	372 (95.4%)

Socioeconomically, Table 1 shows that 47.7% of participants reported a monthly income of <5000 SAR, 34.1% reported an income between 5000 and 10,000 SAR, and 18.2% reported an income exceeding 10,000 SAR. Family size also emerged as a relevant factor, with nearly 40% of participants having 4-6 children, suggesting a potential influence of caregiving responsibilities and associated physical demands on the occurrence of LBP.

Prevalence of LBP

**Figure 1:**
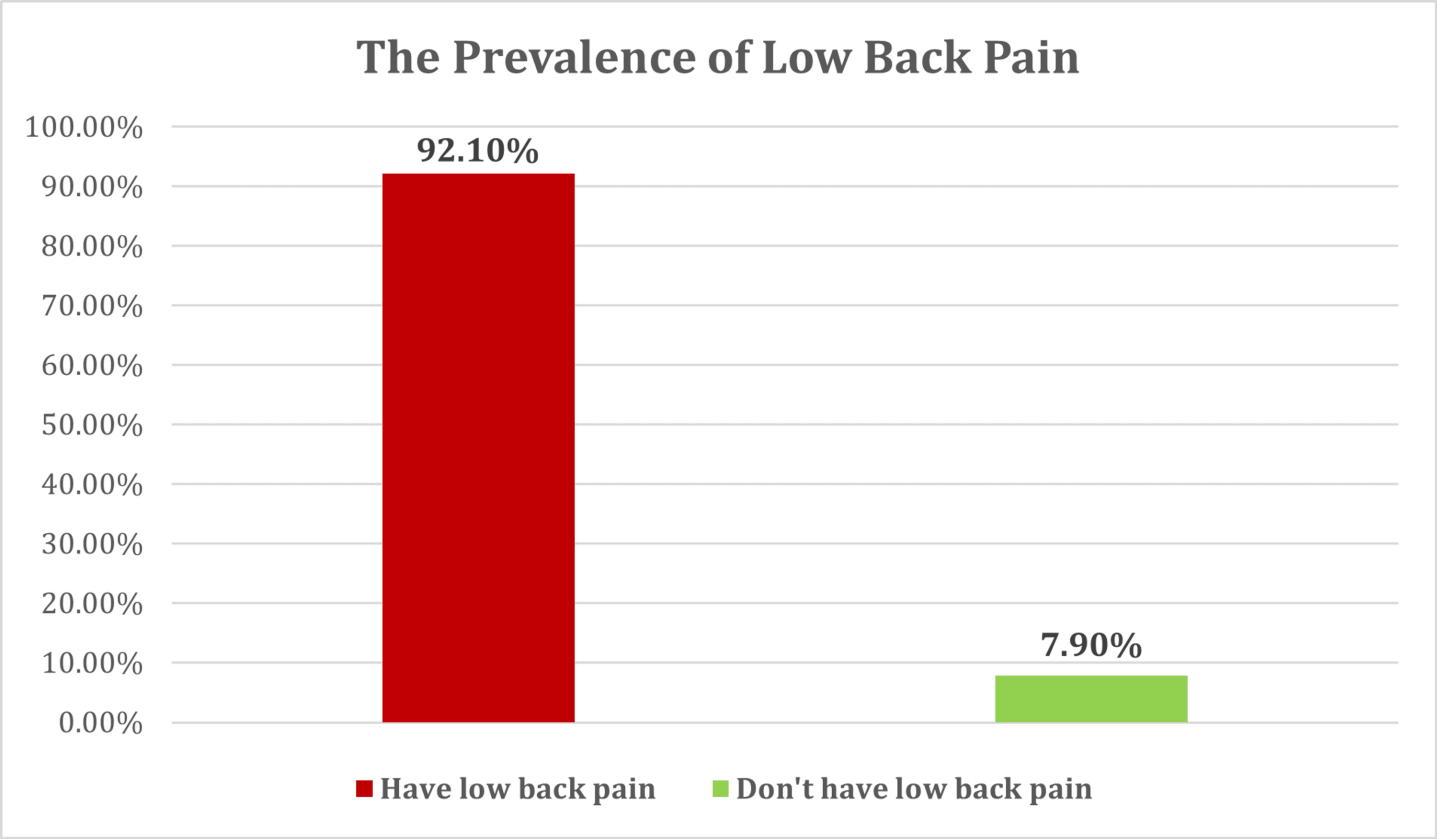
Prevalence of low back pain among nonworking women in the Eastern region, Saudi Arabia

The prevalence of LBP among nonworking women in the Eastern region of Saudi Arabia, as evaluated in this study, was notably high, as shown in Figure 1. A total of 359 participants (92.1%) reported experiencing LBP, while only 31 participants (7.9%) reported not having this condition.

Characteristics of LBP

**Figure 2:**
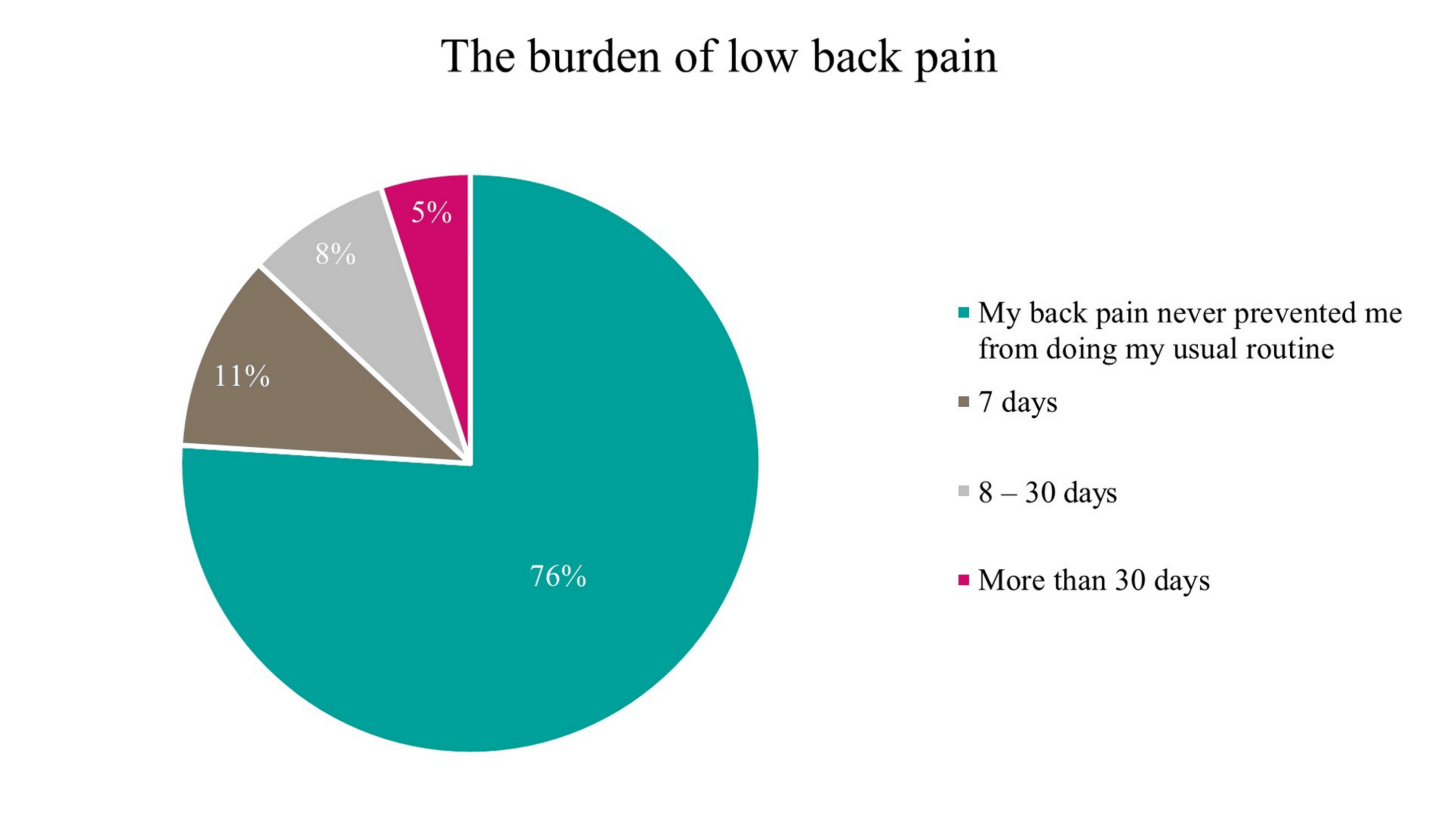
The burden of low back pain among the nonworking women in the Eastern region, Saudi Arabia

As shown in Table 2, the characteristics of LBP reported by participants varied. Stiffness was reported by 42.1% of participants, followed by an unpleasant sensation in 37.6%, numbness in 1.7%, an electrical sensation in 9.2%, and loss of strength in 4.5%. The remaining 5.0% of participants were unable to describe the nature of their pain. Regarding hospitalization history related to LBP, 24.2% of participants reported having been hospitalized, while 75.8% had not. The duration of pain over the preceding 12 months varied: 35.4% experienced pain for up to 7 days, 12.8% for 9-30 days, 32.0% for more than 30 days but not daily, and 19.8% experienced pain daily, as illustrated in Figure 2. Concerning the burden of pain during the past 12 months, 76.0% of participants reported that LBP never prevented them from performing their usual daily activities. Pain occurrence within the last 7 days was reported by 62.7% of participants, whereas 37.3% reported no pain during this period.

**Table 2:** Characteristics of low back pain among nonworking women in the Eastern region, Saudi Arabia

Risk factors	Category	Frequency, n (%)
Nature of pain	Stiffness	151 (42.1%)
	Unpleasant feeling	135 (37.6%)
	Numbness	6 (1.7%)
	Electrical sensation	33 (9.2%)
	Loss of strength	16 (4.5%)
	I can’t describe	18 (5%)
Hospitalization history	Yes	87 (24.2%)
	No	272 (75.8%)
Pain duration (last 12 months)	7 days	127 (35.4%)
	9-30 days	46 (12.8%)
	More than 30 days, but not every day	115 (32%)
	Every day	71 (19.8%)
Pain burden (last 12 months)	Doesn’t affect my usual routine	273 (76%)
	7 days	40 (11.1%)
	8-30 days	29 (8.1%)
	More than 30 days	17 (4.7%)
Pain occurrence (last 7 days)	Yes	225 (62.7%)
	No	134 (37.3%)
Assumed cause of the pain	Trauma or injury	16 (4.5%)
	Sudden movement	34 (9.5%)
	Bad posture	154 (42.9%)
	Other	155 (43.2%)
Onset of the pain	Suddenly	167 (46.5%)
	Gradually	192 (53.5%)
Course of the pain	Intermittent	76 (21.2%)
	Constant	283 (78.8%)
Timing of the pain	Morning	32 (8.9%)
	Noon	7 (1.9%)
	Evening	30 (8.4%)
	Before bedtime	57 (15.9%)
	Whole day	35 (9.7%)
	Any time during day	198 (55.2%)
Associated symptoms		
Unexplained weight loss in past few days	Yes	36 (10%)
	No	323 (90%)
Fever or chills at night	Yes	41 (11.4%)
	No	318 (88.6%)
Progressive or severe weakness in the lower extremities	Yes	58 (16.2%)
	No	301 (83.8%)
Progressive or severe sensory loss in the lower extremities	Yes	74 (20.6%)
	No	285 (79.4%)
Urine incontinence	Yes	58 (16.2%)
	No	301 (83.8%)

Risk factors

Among the nonworking women in the Eastern region of Saudi Arabia, the majority (93.6%) reported that they had never smoked, indicating a minimal contribution of smoking to LBP in this population. A small proportion of participants were former smokers (3.1%) or current smokers (3.3%). With respect to physical exercise, nearly half of the participants (48.2%) reported exercising very infrequently. Equal proportions (15.1% each) reported exercising 1-2 times per week and 3-5 times per week, while only 2.1% exercised more than five times weekly. Additionally, 19.5% reported no engagement in physical exercise.

Daily physical activity duration varied, with 33.8% reporting 30 minutes of activity per day, 19.5% reporting one hour, 6.4% reporting 2-3 hours, and 2.8% reporting more than three hours. Notably, 37.4% did not engage in any daily physical activity. Regarding weight lifting or loading activities, most participants (60.3%) reported never performing such tasks, 34.1% did so occasionally, and 5.6% engaged in these activities regularly. Body mass index (BMI) was distributed across the cohort, with 31.4% classified as underweight, 32.6% as normal weight, 30.1% as overweight, and the remainder classified as obese, indicating that LBP was observed across all BMI categories (Table 3).

**Table 3:** Profile of low back pain risk factors among the nonworking women in the Eastern region, Saudi Arabia

Risk factor		N	%
Smoking	Never smoke	365	93.6
	Former smoker	12	3.1
	Current smoker	13	3.3
Weekly exercise	Very rare	188	48.2
	1-2 a week	59	15.1
	3-5 times a week	59	15.1
	>5 times a week	8	2.1
	I don't practice physical activity	76	19.5
Daily physical activity duration	30 mins	132	33.8
	1 hour	76	19.5
	2-3 hours	25	6.4
	>3 hours	11	2.8
	I don't practice physical activity	146	37.4
Weight lifting/loading	Never	235	60.3
	Sometimes	133	34.1
	Always	22	5.6
Body mass index (BMI)	Underweight	23	5.9
	Healthy weight	122	31.4
	Overweight	127	32.6
	Obese	117	30.1

Associated factors

With regard to the relationship between demographic characteristics and the prevalence of LBP, age was the only variable that showed a statistically significant association (p = 0.034) among nonworking women in the Eastern region of Saudi Arabia. As shown in Table 4, participants in the 36-50-year age group represented the highest proportion of those reporting LBP.

**Table 4:** Association between demographic profile and the prevalence of low back pain among nonworking women in Eastern region, Saudi Arabia *Significant (p < 0.05).

Variable	Category	Lower back pain		χ² (df)	p-value
		No	Yes		
Age	<25	9 (29.0%)	46 (12.8%)	8.543 (3)	0.036*
	26-35	10 (32.3%)	107 (29.8%)		
	36-50	7 (22.6%)	157 (43.7%)		
	More than 50	5 (16.1%)	49 (13.6%)		
Education	Postgraduate	1 (3.2%)	16 (4.5%)	0.165 (3)	0.983
	University	16 (51.6%)	187 (52.1%)		
	Pre-university	13 (41.9%)	147 (40.9%)		
	No education	1 (3.2%)	9 (2.5%)		
Marital status	Single	9 (29.0%)	41 (11.4%)	9.135 (3)	0.028*
	Married	22 (71.0%)	298 (83.0%)		
	Divorced	0 (0.0%)	10 (2.8%)		
	Widowed	0 (0.0%)	10 (2.8%)		
Income	<5000 SAR	16 (51.6%)	170 (47.4%)	2.488 (2)	0.288
	5000-10,000 SAR	7 (22.6%)	126 (35.1%)		
	>10,000 SAR	8 (25.8%)	63 (17.5%)		
Number of children	Zero	9 (30.0%)	61 (17.0%)	5.445 (3)	0.142
	1-3	10 (33.3%)	121 (33.7%)		
	4-6	11 (36.7%)	144 (40.1%)		
	7-10	0 (0.0%)	33 (9.2%)		
Currently pregnant	Yes	1 (3.2%)	17 (4.7%)	0.148 (1)	0.701
	No	30 (96.8%)	342 (95.3%)		

Although Table 2 indicates that LBP affected nonworking women across a wide range of lifestyles and body weight categories, association analysis revealed no statistically significant relationships between LBP and education level (p = 0.954), marital status (p = 0.061), socioeconomic status (p = 0.305), number of children (p = 1.000), or current pregnancy status (p = 0.135).

## Discussion

This study demonstrates a very high prevalence of LBP among nonworking women in the Eastern Province of Saudi Arabia. The overall pattern suggests that symptoms are often persistent, commonly described as stiffness, and most prominent in midlife. Although overweight and obesity were common in this cohort, BMI did not show a statistically significant association with LBP in the present analysis.

The findings confirm the hypothesis of a high prevalence of LBP among nonworking women. Previous studies suggest that women may be biologically predisposed to LBP, particularly during the perimenopausal and menopausal periods, due to hormonal changes, reduced bone density, altered muscle function, and weight gain, all of which may increase mechanical stress on the spine [15,16]. In this study, the highest prevalence of LBP was observed among women aged 36-50 years. This may be related to a combination of physiological changes, lifestyle stressors, sedentary behavior, hormonal fluctuations, and pre-existing musculoskeletal conditions. A substantial proportion of participants with low socioeconomic status (47.4%) reported LBP, consistent with findings from previous studies [17]. Factors such as financial strain, inadequate housing, and limited access to health resources may contribute to the development and persistence of LBP. These factors are also closely linked to psychological stress, which has been associated with increased muscle tension and heightened pain perception [18-23].

Married participants were more likely to report LBP than unmarried participants, possibly reflecting increased household responsibilities, reduced opportunities for self-care, and greater emotional and physical demands. In addition, a large proportion of participants with LBP (39.8%) had four to six children, consistent with prior studies reporting a higher prevalence of LBP among women with multiple pregnancies [24]. This finding reflects the relatively larger family size among nonworking women in this region and suggests a potential cumulative physical burden related to childcare and domestic activities. Most participants with LBP (78.8%) reported persistent symptoms, with 19.8% experiencing daily pain over the preceding year. Despite this, 75.8% had no history of hospitalization for LBP. This suggests that many women may rely on self-management strategies, such as rest, home remedies, or alternative therapies, and may not seek hospital care unless symptoms become severe or disabling.

Notably, most participants with LBP (76%) reported that their pain did not interfere with their usual daily activities. This contrasts with findings from other studies that reported greater functional limitation among housewives with chronic or recurrent LBP [9]. Smoking did not show a significant association with LBP in the present study, consistent with several previous reports [14,15], although other studies have identified smoking as a risk factor for LBP [25]. Although a large proportion of participants who reported LBP were overweight (32.6%) or obese (31.5%), BMI was not significantly associated with LBP in this cohort. This finding is consistent with some studies that reported no difference in BMI between individuals with and without LBP [26], although other studies have demonstrated a positive association between higher BMI and LBP [14,15,24]. Weight reduction and lifestyle modification may nonetheless be beneficial, as excess body weight can increase spinal load and negatively affect musculoskeletal health. Physical inactivity was common in this population, with nearly half of participants reporting infrequent exercise and over one-third reporting no regular physical activity. Sedentary behavior may increase susceptibility to LBP by contributing to muscle weakness and poor postural control. Regular physical activity is known to strengthen supporting musculature and improve spinal stability, potentially reducing LBP risk. However, further studies are needed to clarify the role of exercise in this specific population. Prior research has identified a lack of regular physical activity as a risk factor for LBP in other groups, including healthcare workers [27].

Understanding the prevalence and associated factors of LBP in specific populations is essential for effective prevention and management strategies. This study highlights the potential contribution of poor sitting habits to LBP, as reported in earlier literature [27]. Public health initiatives that promote ergonomic awareness, physical activity, and healthy lifestyle practices may help reduce the burden of LBP and improve the quality of life among nonworking women. Several limitations should be acknowledged. The absence of a psychological assessment limits the evaluation of psychosocial contributors to LBP. Coexisting medical conditions were not analyzed, which may have influenced pain prevalence. The relatively limited sample size restricts generalizability and may increase the risk of selection bias. Sleep patterns and sleep disorders, which may have a bidirectional relationship with LBP, were not assessed. Finally, the use of an online, self-administered questionnaire introduces potential sampling and self-report bias, including recall bias and social desirability bias. Future studies should address these limitations by incorporating psychological variables, medical comorbidities, objective measures, and larger, more representative samples to further elucidate the determinants of LBP in this population.

## Conclusions

LBP appears to be highly prevalent among nonworking women in the Eastern Province of Saudi Arabia, with age emerging as the only factor significantly associated with LBP in this sample. Notably, many women reported continuing their usual daily activities despite symptoms, suggesting that LBP in this group may be underrecognized and underaddressed rather than absent. These findings highlight the need for proactive community awareness, early assessment, and access to conservative management strategies, including education, exercise guidance, and ergonomic advice, particularly for women in midlife. Given the cross-sectional, self-reported, online design of this study, future research should incorporate validated pain and disability measures and evaluate healthcare-seeking behavior, psychosocial factors, and comorbidities to better define functional impact and identify modifiable contributors.

## Appendices

استبيان قياس مدى انتشار آلام أسفل الظهر لدى ربات البيوت غير العاملات في المنطقة الشرقية بالمملكة العربية السعودية

: البيانات الشخصية

هل تعمل؟

☐ نعم

☐ لا

الجنس:

☐ أنثى

☐ ذكر

العمر:

☐ أقل من ٢٥

☐ من ‏٢٦‏ إلى ‏٣٥‏

☐ من ‏٣٦‏ إلى ‏٥٠‏

☐ أكثر من ٥٠

التعليم:

☐ الدراسات العليا

☐ الجامعة

☐ ما قبل الجامعة

☐ لا تعليم

الحالة الزوجية:

☐ عزباء

☐ متزوجة

☐ متطلقة

☐ أرملة

الوضع الاجتماعي والاقتصادي (الدخل الشهري):

☐ أقل من 5000 ريال سعودي

☐ من 5000 إلى 10,000 ريال سعودي

☐ أكثر من 10,000 ريال سعودي

 المنطقة:

☐ المنطقة الشرقية بالمملكة العربية السعودية

☐ خارج المنطقة الشرقية بالمملكة العربية السعودية

الجنسية:

☐ سعودية

☐ غير سعودية

عدد الأطفال:

______________________________

عدد حالات الحمل:

______________________________

هل أنتِ حامل حاليًا؟

☐ نعم

☐ لا

: عوامل الخطر

:التدخين

☐ غير مدخن

☐ مدخن سابق

☐ مدخن حالي

كم مرة تتمرن في الأسبوع؟

☐ نادر جدًا

☐ من ‏١‏ إلى ‏٢‏ مرة في الأسبوع

☐ من ‏٣‏ إلى ‏٥‏ مرات في الأسبوع

☐ أكثر من ٥ مرات في الأسبوع

☐ أنا لا أمارس أي نشاط بدني

ما هي المدة التي تمارس فيها النشاط البدني؟

☐ ٣٠ دقيقة

☐ ساعة واحدة

☐ من ‏٢‏ إلى ‏٣‏ ساعات

☐ أكثر من ٣ ساعات

☐ أنا لا أمارس أي نشاط بدني

رفع/حمل أثقال:

☐ أبدًا

☐ أحيانًا

☐ دائمًا

 الوزن (kg):

______________________________

الطول (cm):

______________________________

: آلام أسفل الظهر

هل سبق لك أن عانيتِ من مشاكل في أسفل الظهر ألم أو عدم راحة؟

☐ نعم

☐ لا

كيف تصفين طبيعة شكواك؟

☐ شعور مزعج

☐ تيبّس/تصلّب

☐ خدر

☐ فقدان القوة

☐ الإحساس الكهربائي

☐ في الوقت الحالي، لا أستطيع أن أصف

هل سبق لك أن دخلتِ المستشفى بسبب مشاكل في أسفل الظهر؟

☐ نعم

☐ لا

ما هي المدة الزمنية التي عانيتِ فيها من مشاكل في أسفل الظهر خلال الأشهر الـ 12 الماضية؟

☐ ٧ أيام

☐ من ‏٨‏ إلى ‏٣٠‏ يومًا

☐ أكثر من ٣٠ يومًا، ولكن ليس كل يوم

☐ كل يوم

ما هي المدة الإجمالية التي منعتك فيها مشكلة أسفل الظهر من القيام بعملك العادي في المنزل أو بعيداً عن المنزل خلال الأشهر الـ 12 الماضية؟

☐ لم يمنعني ألم الظهر أبدًا من القيام بروتيني المعتاد

☐ ٧ أيام

☐ من ‏٨‏ إلى ‏٣٠‏ يومًا

☐ أكثر من ٣٠ يومًا

هل عانيتِ من مشاكل في الظهر في أي وقت خلال آخر 7 أيام؟

☐ نعم

☐ لا ما هو سبب ألم الظهر برأيك؟

☐ صدمة أو إصابة

☐ حركة مفاجئة

☐ وضعية سيئة

☐ أخرى هل بدأ الألم؟

☐ فجأة

☐ تدريجياً يعد ألمك:

☐ ثابت

☐ متقطع متى تشعرين بالألم خلال اليوم؟

☐ صباحاً

☐ وقت الظهر

☐ مساءاً

☐ قبل وقت النوم

☐ طوال اليوم

☐ في أي وقت خلال اليوم هل عانيتِ من فقدان الوزن غير المبرر في الأيام القليلة الماضية؟

☐ نعم

☐ لا هل تعانين من أي حمى أو قشعريرة في الليل؟

☐ نعم

☐ لا هل تعانين من أي فقدان حسي تدريجي أو شديد في الأطراف السفلية؟

☐ نعم

☐ لا هل تعانين من أي ضعف تدريجي أو شديد في الأطراف السفلية؟

☐ نعم

☐ لا هل تعانين من سلس البول؟

☐ نعم

☐ لا نهاية الإستبيان

Questionnaire: prevalence of low back pain among nonworking housewives in the Eastern Province, Kingdom of Saudi Arabia

Personal information

Do you work?

☐ Yes

☐ No

Sex:

☐ Female

☐ Male

Age:

☐ Under 25 year old

☐ 26-35 year old

☐ 36-50 year old

☐ Over 50 year old

Education:

☐ Postgraduate studies

☐ University

☐ Pre-university

☐ No education

Marital status:

☐ Single

☐ Married

☐ Divorced

☐ Widowed

Socioeconomic status (monthly income):

☐ Less than 5000 SAR

☐ 5000 to 10,000 SAR

☐ More than 10,000 SAR

Region:

☐ Eastern Province of Saudi Arabia

☐ Outside the Eastern Province of Saudi Arabia

Nationality:

☐ Saudi

☐ Non-Saudi

Number of children: ____________________________

Number of pregnancies: ____________________________

Are you currently pregnant?

☐ Yes

☐ No

Risk factors

Smoking:

☐ Non-smoker

☐ Former smoker

☐ Current smoker

How many times do you exercise per week?

☐ Very rarely

☐ 1-2 times per week

☐ 3-5 times per week

☐ More than 5 times per week

☐ I do not do any physical activity

How long do you do physical activity (per session)?

☐ 30 minutes

☐ 1 hour

☐ 2-3 hours

☐ More than 3 hours

☐ I do not do any physical activity

Lifting/carrying heavy objects:

☐ Never

☐ Rarely

☐ Sometimes

☐ Always

Weight (kg): ____________________________

Height (cm): ____________________________

Low back pain

Have you ever had low back problems (pain or discomfort)?

☐ Yes

☐ No

How would you describe your complaint?

☐ An uncomfortable sensation

☐ Stiffness/tightness

☐ Numbness

☐ Loss of strength

☐ Electric/shock-like sensation

☐ Currently, I cannot describe it

Have you ever been hospitalized because of low back problems?

☐ Yes

☐ No

How long did you have low back problems during the past 12 months?

☐ 7 days

☐ 8-30 days

☐ More than 30 days, but not every day

☐ Every day

In total, how long did your low back problem prevent you from doing your usual work at home or outside the home during the past 12 months?

☐ Back pain never prevented me from my usual routine

☐ 7 days

☐ 8-30 days

☐ More than 30 days

Have you had back problems at any time during the last 7 days?

☐ Yes

☐ No

What do you think is the cause of your back pain?

☐ Trauma or injury

☐ Sudden movement

☐ Poor posture

☐ Other

How did the pain start?

☐ Suddenly

☐ Gradually Your pain is:

☐ Constant

☐ Intermittent

When do you feel pain during the day?

☐ Morning

☐ Noon/midday

☐ Evening

☐ Before bedtime

☐ All day

☐ At any time during the day

Have you had unexplained weight loss in the past few days?

☐ Yes

☐ No

Do you have any fever or night chills?

☐ Yes

☐ No

Do you have any progressive or severe loss of sensation in the lower limbs?

☐ Yes

☐ No

Do you have any progressive or severe weakness in the lower limbs?

☐ Yes

☐ No

Do you have urinary incontinence?

☐ Yes

☐ No
